# Protective Effect of Green Tea (*Camellia sinensis* (L.) Kuntze) against Prostate Cancer: From In Vitro Data to Algerian Patients

**DOI:** 10.1155/2017/1691568

**Published:** 2017-01-04

**Authors:** Somia Lassed, Cláudia M. Deus, Radja Djebbari, Djamila Zama, Paulo J. Oliveira, Albert A. Rizvanov, Abderrezak Dahdouh, Fadila Benayache, Samir Benayache

**Affiliations:** ^1^Unité de Recherche Valorisation des Ressources Naturelles, Molécules Bioactives et Analyses Physicochimiques et Biologiques (VARENBIOMOL), Université Constantine 1, 25000 Constantine, Algeria; ^2^Laboratoire de Physiologie Animale, Département de Biologie Animale, Faculté des Sciences de la Nature et de la Vie, Université Constantine 1, 25000 Constantine, Algeria; ^3^Clinic of Urology-Nephrology and Kidney Transplant Daksi, Constantine, Algeria; ^4^CNC–Center for Neuroscience and Cellular Biology, UC-Biotech Building, Biocant Park, University of Coimbra, Coimbra, Portugal; ^5^Institute of Fundamental Medicine and Biology, Kazan Federal University, Kazan, Russia

## Abstract

Green tea (GT) has been studied for its effects as antioxidant and cancer-preventive agent. Epidemiological studies showed that GT consumption decreases the risk for prostate cancer (PC). To investigate whether erythrocyte oxidative stress (OS) is associated with PC and whether daily consumption of GT improves the oxidative phenotype, we performed a study in a group of Algerian PC patients, preceded by an in vitro study to characterize composition and antioxidant/antiproliferative activities of the GT used. This contained a high content of phenolic and flavonoid compounds, demonstrating in vitro antioxidant activity and significant antiproliferative effect on human prostate cancer PC-3 cell line. Seventy PC patients and 120 age-matched healthy subjects participated in the study, with glutathione (GSH), malondialdehyde (MDA), and catalase activity evaluated before and after GT consumption. The results showed a reduced GSH and catalase activity and a high level of MDA in erythrocytes from PC patients. The consumption of 2-3 cups per day of GT during 6 months significantly increased GSH concentration and catalase activity and decreased MDA concentration. In conclusion, GT significantly decreased OS in Algerian PC patients. Regular consumption of GT for a long period may prevent men from developing PC or at least delay its progression.

## 1. Introduction

Prostate cancer (PC) is the second most common malignancy diagnosed in men and the fifth leading cause of mortality in the world. In fact, PC was one of the most frequently diagnosed male neoplasias and the sixth leading cause of death in Algeria in 2012 [1]. As in most cancers, the etiological factors of PC still remain poorly understood although many studies suggested that aging [2, 3], diet [4], and inflammation [5] are involved in PC development and progression, with oxidative stress possibly being a common link. In fact, several studies in humans showed significant alterations in oxidant-antioxidant balance in PC patients when compared to controls. Significant high levels of malondialdehyde, ceruloplasmin, and lower levels of reduced glutathione and glutathione peroxidase, catalase, and superoxide dismutase activities were observed in PC patient blood [6–8].

Green tea, a beverage prepared from the dry leaves of* Camellia sinensis* (L.) Kuntze, has been extensively studied for its effect as a potent antioxidant [9, 10] and on cancer prevention [11–13]. Epidemiological studies found that green tea consumption may decrease the risk for PC [14, 15]. Using PC cell lines, it was demonstrated that green tea polyphenols, especially catechins, the major species, inhibit carcinogenesis through different mechanisms of action including induction of cell cycle arrest [16], apoptosis [17], inhibition of the insulin-like growth factor receptor [18] and androgen receptor downregulation by interaction with its ligand-binding domain [19]. In many preclinical trials, the administration of an oral infusion of green tea catechins in TRAMP (transgenic adenocarcinoma of mouse prostate) mice confirmed the efficacy of green tea in decreasing PC progression [20, 21]. However, few studies were performed in human [22–24] with only a few finding encouraging results [22, 24].

The current study aimed to investigate whether PC was associated with increased oxidative stress in erythrocytes in a set of Algerian patients and whether green tea intake inversely correlated with oxidative stress, a possible factor involved in PC development and progression. We selected a popular type of commercial Chinese green tea in Algeria and measured its phenol and flavonoid content, as well as antioxidant and antiproliferative activities in vitro. In the follow-up translational step, we evaluated oxidative stress markers in peripheral blood in Algerian PC patients before and after green tea consumption.

## 2. Materials and Methods

### 2.1. Materials

Dulbecco's modified Eagle's medium (DMEM, D5648), Roswell Park Memorial Institute medium (RPMI, 1640), penicillin, streptomycin, fetal bovine serum (FBS), and 0.25% Trypsin-EDTA were purchased from Gibco-Invitrogen (Grand Island, NY). Sodium chloride (NaCl), sulforhodamine B sodium salt (SRB), Tris, and trypan-blue solution were obtained from Sigma-Aldrich Chemical Co. (Saint Louis, MO, USA). Ellman's Reagent (DTNB, D8130), thiobarbituric acid (TBA, T5500), L-glutathione reduced (GSH, G4251) were purchased from Sigma-Aldrich (St. Louis, MO, USA). All other reagents and chemical compounds used were of the greatest degree of purity commercially available. In the preparation of every solution, including buffers, ultrapure distilled water (conductivity < 18 *μ*S·cm^−1^), filtered by the Milli-Q from a Millipore (Billerica, MA) system, was always used in order to minimize as much as possible contamination with metal ions.

### 2.2. Experimental Approach: In Vitro

#### 2.2.1. Green Tea Extraction

Two thousand grams of commercial Chinese green tea leaves of* Camellia sinensis* (L.) Kuntze plant was macerated with EtOH/H_2_O (7 : 3 v/v) for 48 h three successive times. The combined filtrate was dried by evaporation and the ethanolic extract obtained was solubilized in 800 mL H_2_O. The aqueous filtrate was successively extracted with chloroform (CHCl_3_), ethyl acetate (EtOAc), and* n*-butanol (*n*-BuOH). The organic phases were concentrated in vacuum to obtain the following extracts: (CHCl_3_) (4.7 g), EtOAc (27.66 g), and* n*-BuOH (46.31 g).

#### 2.2.2. Determination of Total Phenolic and Flavonoid Content

The total phenolic content in green tea extracts (CHCl_3_, EtOAc, and* n*-BuOH) was measured using the method of Singleton et al. [25]. To 20 *μ*l of each green tea extract prepared in methanol (1 mg/ml), 100 *μ*l of Folin-Ciocalteu and 1580 *μ*l of distilled water were added successively, flowed three min later by 300 *μ*l of sodium carbonate (20%). Samples were stirred for 2 h at room temperature, and the absorbance was then evaluated at 765 nm. The concentration of total phenolic compounds was determined as *μ*g of gallic acid equivalent (GAE) per mg of extract using a standard curve which was prepared using gallic acid solutions (0 to 500 mg/ml) solubilized in MeOH/H_2_O (1 : 9 v/v).

The total flavonoid content in green tea extracts was determined according to the method of Wang et al. [26]. 0.5 ml of 2% AlCl_3_ was mixed with 0.5 ml of sample. After 1 h incubation at room temperature, the absorbance was measured at 420 nm. The concentration of flavonoids was determined as *μ*g of quercetin equivalent (QE) per mg of extract using standard quercetin calibration curve.

#### 2.2.3. HPLC-TOF/MS Analysis of Green Tea Extracts

To analyze the phenolic content of the different green tea extracts, high-performance liquid chromatography/time-of-flight mass spectrometry (HPLC-TOF/MS) analysis was used. Agilent Technologies 1260 Infinity HPLC System was coupled to a 6210 time-of-flight (TOF) LC/MS detector and ZORBAX SB-C18 (4,6 × 100 mm, 3.5 *μ*m) column. The mobile phases A and B were ultrapure water solution with 0.1% formic acid and acetonitrile, respectively. Flow rate was 0.6 ml/min and column temperature was 35°C. The green tea extracts (200 ppm) and stock solutions of 23 standard phenolic compounds (2.5 ppm) were prepared in methanol at room temperature. The samples were filtered passing through a PTFE (0.45 *μ*m) filter by an injector to remove particulates. The injection volume was 10 ml and the solvent program was as follows: 0 min 10% B; 0-1 min 10% B; 1–20 min 50% B; 20–23 min 80% B; 23–25 min 10% B; 25–30 min 10% B. The ionization mode of MS-TOF instrument was ES negative with gas temperature of 325°C, gas flow of 10.0 l/min, and nebulizer of 40 (psi). The phenolic content of green tea extracts was determined by comparing retention times and* m/z* values of green tea extracts and standard phenolic compounds.

#### 2.2.4. Evaluation of the Antioxidant Activity

(*1) DPPH Radical-Scavenging Activity Assay*. The ability of green tea extracts to quench DPPH (2,2-diphenyl-1-picrylhydrazyl) radical was evaluated by the method of Braca et al. [27]. To increasing concentrations (1, 2.5, 5, 10, 20, and 25 *μ*g/ml) of methanol-dissolved extracts, 3 ml of methanol DPPH solution (0.004%) was added. Test tubes were incubated at room temperature for 30 min, and absorbance was then measured at 517 nm. Tests were carried out in triplicate and ascorbic acid was used as positive control. The percentage of DPPH scavenging activity (*I*%) was calculated using the following equation (1):

(1) $$\begin{array}{c} I\%=\left[\;\frac{\left(A_{0}-A_{1}\right)}{A_{0}}\;\right]\times 100, \end{array}$$

where *A*_0_ is the absorbance of DPPH solution alone and *A*_1_ is the absorbance of DPPH solution + extract or vitamin C. The half inhibition concentration (IC_50_) of green tea extracts was calculated from the plot of percentage of inhibition against concentration of green tea extracts.

*(2) Inhibition of Lipid Peroxidation*. The lipid peroxidation assay was performed according to the modified protocol of Cao and Ikeda using egg* vitellose* [28]. To evaluate the capacity of green tea extracts to inhibit lipid peroxidation, 0.5 ml of 10% egg* vitellose* homogenate as lipid-rich media was mixed with 50 *μ*l of FeSO_4_ (0.07 M) and then incubated with increasing concentrations of green tea extracts or vitamin C at 37°C for 30 min. After incubation, 1 ml TCA 20% (trichloroacetic acid) and 1.5 ml TBA 1% (thiobarbituric acid) were successively added. The samples were mixed and then heated for 15 min at 95°C. After centrifugation (400*g* for 20 min), the resulting thiobarbituric reacting substances (TBARS) were measured in the supernatant at 532 nm. The lipid peroxidation inhibition was calculated as percentage (*I*%) according to (1), where *A*_0_ is the absorbance of the control (without extract or vitamin C) and *A*_1_ is the absorbance of sample + extract or vitamin C.

#### 2.2.5. Evaluation of the Cytotoxicity Effect of Green Tea Extracts

*(1) Cell Culture and Treatments*. The human metastatic prostate cancer cell line PC-3 [29] and human foreskin BJ fibroblasts [30], purchased from America Tissue Type Collection (Manassas, VA), were cultured in RPMI (1640) and DMEM (D5648) media, purchased from Gibco-Invitrogen (Grand Island, NY), supplemented with 1.5 g/l sodium bicarbonate, 10% fetal bovine serum, 100 U/ml of penicillin, and 100 *μ*g/ml of streptomycin in tissue-culture dishes at 37°C in a humidified atmosphere of 5% CO_2_. All cells were passaged by trypsinization when reaching 70–80% confluence and all experiments were performed in log-phase growth cultures. Green tea extracts (ChCl_3_, EtOAc, and *n*-BuOH) were prepared in dimethyl sulfoxide (DMSO) and stored at 4°C in the dark. The total volume of DMSO was always smaller than 0.1%, which had negligible effects in all experiments. Green tea extracts were directly added to the culture medium at the described concentrations. Vehicle controls received an equivalent amount of DMSO only.

*(2) Cell Proliferation Measurements*. The sulforhodamine B (SRB) assay was used to measure cell protein, which is dependent on the amount of cells in each well [31]. In the present study, we used both human foreskin BJ fibroblasts, which have a long lifespan and are commonly used as a nontumor control cell line [32, 33] and the human PC-3 cell line, commonly used as an in vitro model for PC studies [29]. Both cell lines were seeded in 48-well plates with a final volume of 500 *μ*l per well at a density of 10,000 and 20,000 cells per ml, respectively. The two cell lines were treated with increasing concentrations of green tea extracts (5, 10, 25, and 50 *μ*g/ml). Twenty-four hours after drug addition, the incubation medium was removed, wells rinsed with 1% PBS, and cells were fixed in 1% acetic acid in ice-cold methanol for at least one day. Cells were then incubated with 0.05% (w/v) SRB reagent dissolved in 1% acetic acid for 1 h at 37°C. Unbound dye was removed with 1% acetic acid. Dye bound to cell proteins was extracted with 10 mM Tris-base solution, pH 10. After SRB labeling, absorbance was measured at 540 nm in a plate reader and the amount of dye released which is proportional to the number of cells present in the dish was measured [31]. The results were expressed as a percentage of control.

### 2.3. Human Studies

#### 2.3.1. Study Subjects

Ninety patients diagnosed with histologically confirmed PC at the Clinic of Urology-Nephrology and Kidney Transplant Daksi, Constantine, Algeria, were interviewed and invited to participate in the study. Patients received oral and written information about the study and gave their written consent. Three patients refused to participate. Eighty-seven volunteers received the same brand of green tea after the evaluation of its antioxidant and cytotoxic effect (in vitro approach). The offered quantity of green tea was weighed and divided into doses of 2 g for each. Subjects were asked to drink 5 cups of green tea infusion per day for 6 months. Green tea was prepared every day at the same temperature (70–80°C), time of infusion (5 minutes), and concentration (2 g of tea leaves in 100 ml of water for each cup) [34]. The information about total Gleason score, serum PSA level at diagnosis, and primary treatment taken for patients were obtained from the medical record. No smoking or alcohol drinking age-matched 120 healthy subjects were selected carefully in the Clinic of Urology-Nephrology and Kidney Transplant Daksi, Constantine, Algeria, and in the blood sample collection room of the Laboratory of Biochemistry of Establishment Public Hospital of Chelghoum Laid City, Algeria. Controls were divided into two groups: green tea drinkers (*n* = 35) and nontea drinkers (*n* = 85). The Ethics Committee of the EHS Daksi certified that the data collection was performed at the Department of Urology and Renal Transplant without any risk for patients.

#### 2.3.2. Samples Collection and Preparation of Hemolysates

Heparinized venous blood samples were collected after an overnight fast. Patients sample collection was at the beginning (*T* = 0), after green tea consumption (*T* = 3 months) (*T* = 6 months). Blood samples were immediately centrifuged at 300*g* for 15 min and then serum aliquots were removed and stored at −80°C until assayed. Erythrocytes were washed three times by centrifugation (300*g*, 15 min) in an ice-cold isotonic sodium chloride solution (1 : 10, v/v). The supernatant and buffy coat of white cells was carefully removed after each wash. A portion of the erythrocytes obtained were used for GSH determination, while the remaining was resuspended in the washing solution to give a 50% suspension. Hemolysis of the washed cell suspension was achieved by mixing 1 volume of cells with 9 volumes of cold distilled water. After removing the cell debris by centrifugation (300*g*, 15 min), the hemolysate obtained was used for determination of enzymatic activity of CAT and TBARS measurement. The hemoglobin content in the red blood cell lysate was measured according to the cyanmethemoglobin method using Drabkin's reagent [35].

#### 2.3.3. Biochemical Assays

The GSH concentration was measured using the method described by Beutler et al. [36]. Briefly, 0.2 ml fresh erythrocytes pellet was added to 1.8 ml distilled water followed by 3 ml precipitating solution (1.65 g metaphosphoric acid, 0.2 g EDTA, and 30 g NaCl in 100 ml distilled water). The mixture was allowed to stand 5 min and centrifuged next (300*g*, 15 min). Eight ml of 0.3 mM disodium phosphate solution and 1 ml of DTNB were added to 2 ml of supernatant. A blank was prepared replacing erythrocytes for 0.2 ml of distilled water. GSH standard was prepared with 0.2 ml the glutathione solution, 8 ml of 0.3 mM disodium phosphate solution, and 1 ml of DTNB. The optical density was measured at 412 nm in a spectrophotometer.

Erythrocytes catalase (EC 1.11.1.6) activity was estimated in the hemolysate following the method of Greenwald (1985) [37] based on the scavenging of hydrogen peroxide (0.019 M) in the presence of phosphate buffer (0.1 M, pH 7.4) by catalase present in the sample. Catalase activity was expressed as international unit per g hemoglobin (UI/gHb).

Lipid peroxidation in erythrocytes was evaluated by measuring malondialdehyde (MDA) in the hemolysate according to the double heating method (Draper and Hadley (1990)) [38]. This method is based on the reaction of lipid peroxides (MDA) with thiobarbituric acid (TBA, 0.67%) in acidic environment (TCA, 10%) at 90–100°C to form a pink pigment with absorption maximum at 532 nm. The concentration of MDA was calculated by the absorbance coefficient of MDA-TBA complex 1.56 × 10^5^ cm^−1^ M^−1^ and was expressed in *μ*mol/gHb.

## 3. Statistical Analysis

Sulforhodamine B results were expressed as mean ± SEM and multiple comparisons were performed using one-way analysis of variance (ANOVA) followed by Dunnett's multiple comparisons test. Data from human studies were expressed as mean ± SD and differences between the different groups were using a Student's *t* test. Significance was accepted with *p* < 0.05.

## 4. Results 

### 4.1. In Vitro Characterization of Green Tea Composition and Activity

#### 4.1.1. Total Phenolic and Flavonoid Content of Green Tea Extracts

**Figure 1:**
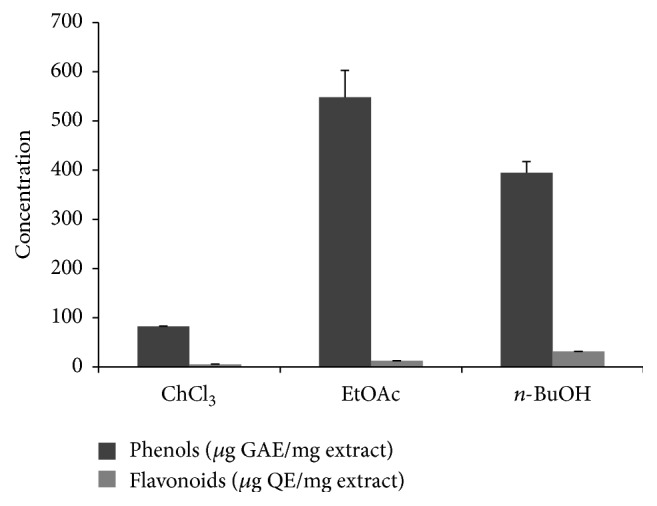
Total phenolic and flavonoid content of green tea extracts, measured as described in Materials and Methods. Concentrations of total phenolic compounds are expressed as *μ*g of gallic acid equivalent (GAE) per mg of extract and total flavonoids are expressed as *μ*g of quercetin equivalent (QE) per mg of extract. Values are means ± SD (*n* = 3).

Total phenolic and flavonoid content in the different green tea extracts obtained showed a high concentration of phenolic and flavonoid compounds especially in the EtOAc and* n*-BuOH extracts. The latter contained 548.33 ± 54.62 and 394.66 ± 22.67 *μ*g of gallic acid equivalent/mg extract of phenolic compounds and 12.16 ± 0.01 and 31.20 ± 0.1 *μ*g of quercetin equivalent/mg extract of flavonoid compounds, respectively. The CHCl_3_ extract contained minor amounts of phenolic and flavonoid compounds, namely, 82.33 ± 5.04 *μ*g of gallic acid equivalent/mg extract of phenolic compounds and 5.04 ± 0.59 *μ*g of quercetin equivalent/mg extract of flavonoid compounds (Figure 1).

#### 4.1.2. Evaluation of Phenolic Content of Green Tea Extracts Using HPLC-TOF/MS

**Figure 2:**
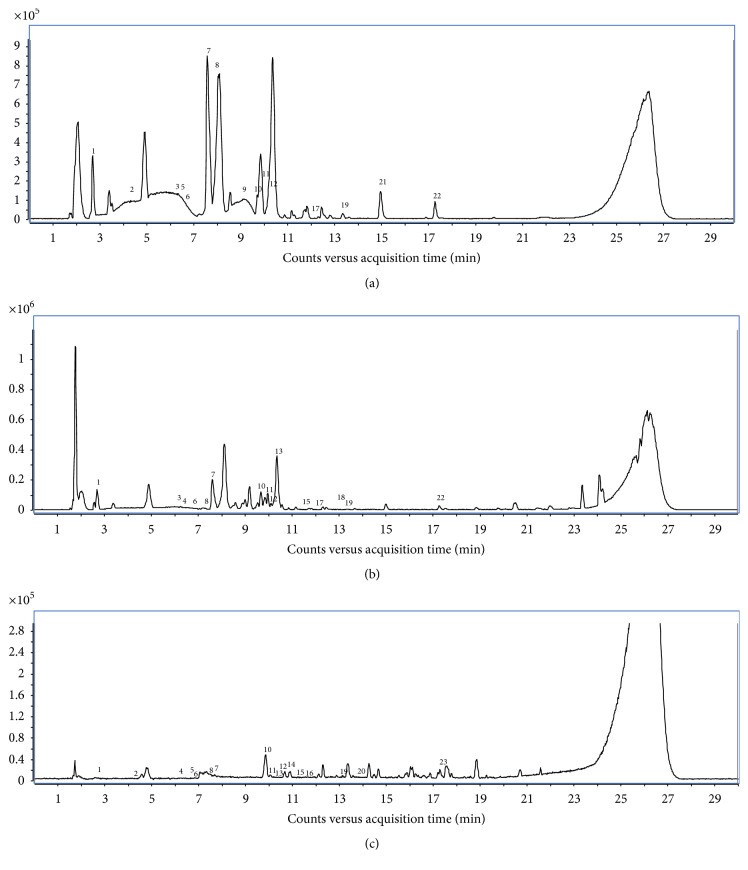
Chromatograms of green tea extracts. (a) Chromatogram of chloroformic (CHCl_3_) green tea extract. (b) Chromatogram of ethyl acetate (EtOAc) green tea extract. (c) Chromatogram of* n*-butanol (*n*-BuOH) green tea extract. The chromatographic conditions were described in Materials and Methods. The phenols detected by HPLC-TOF/MS analysis are expressed as numbers from 1 to 23.

Analysis by HPLC-TOF/MS revealed the presence of different phenols in the different green tea extracts. Using 23 standard phenolic compounds, seventeen different phenols were detected in the EtOAc and* n*-BuOH extracts. A high amount of gallic acid and catechins and relevant amounts of vanillic acid, salicylic acid, rutin, and* p*-coumaric acid were found in the EtOAc extract. The* n*-BuOH extract contains also a high amount of gallic acid followed by rutin, gentisic acid, salicylic acid, and chlorogenic acid. Fifteen phenols were detected in the CHCl_3_ extract although all present in small amounts (Figure 2, Table 1).

#### 4.1.3. Antioxidant Activity of Green Tea Extracts

**Figure 3:**
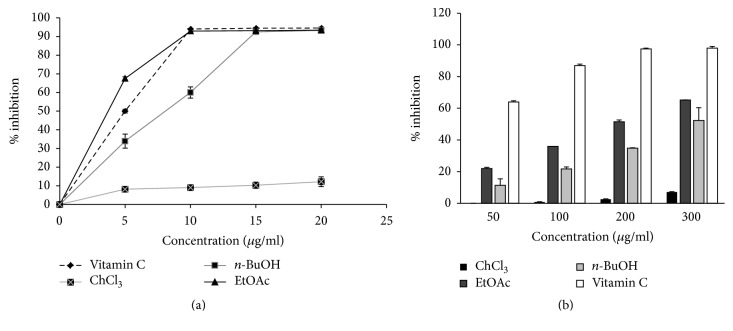
Antioxidant activity of green tea extracts. (a) DPPH scavenging activities of green tea extracts and vitamin C, measured as described in Materials and Methods. (b) Effect of green tea extracts and vitamin C on inhibition of FeSO_4_-induced lipid peroxidation of egg* vitellose*, measured as previously described in Materials and Methods. Values are mean ± SD (*n* = 3).

No significant antioxidant activity was observed for the CHCl_3_ extract but significant effects were found with EtOAc and* n*-BuOH extracts. Compared to vitamin C (IC_50_ = 5 ± 0.1 *μ*g/ml), EtOAc and* n*-BuOH extracts also significantly quenched the DPPH radical (IC_50_ = 2.98 ± 0.32 and 7.58 ± 0.74, resp.) and their effects were dose-dependent. The highest percentages of DPPH inhibition were, respectively, 93.4% and 93.2%, which were similar to vitamin C in the same concentration (20 *μ*g/mL) (Figure 3(a), Table 2).

A dose-dependent decrease in lipid peroxidation was also observed with EtOAc (IC_50_ = 201.01 ± 2.55 *μ*g/ml) and* n*-BuOH (IC_50_ = 302.18 ± 28.31 *μ*g/ml) extracts, although the effects were not comparable to those of vitamin C (IC_50_ = 20 ± 1.06 *μ*g/ml) (Figure 3(b), Table 2).

#### 4.1.4. Cytotoxicity of Green Tea Extracts on Human Prostate Cancer PC-3 Cell Line

**Figure 4:**
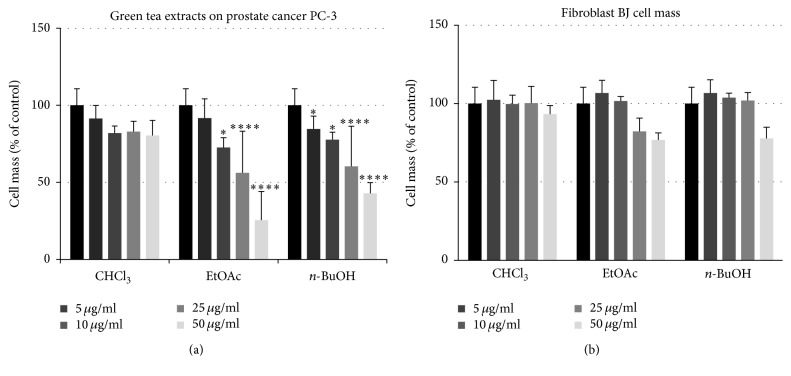
Effects of green tea extracts on prostate cancer PC-3 and fibroblast BJ cell mass. The control value (vehicle only) was determined as 100% to account for the differential proliferation of cell lines. Data are expressed as the means ± SEM of four different experiments, ^*∗*^*p* < 0.05, ^*∗∗∗∗*^*p* < 0.0001 versus control, nontreated cells.

The results regarding cytotoxicity activity using the SRB assay indicated that green tea extracts showed significant antiproliferative activity against the human prostate cancer PC-3 cell line. Dose-dependent toxicity on that cell line was observed after 24 h treatment with EtOAc and* n*-BuOH extracts, with IC_50_ values of 36.37 and 37.74 *μ*g/ml, respectively. However, no effects were observed on the nontumor, control fibroblast BJ cell line with both extracts. No cytotoxicity effects were observed in any of the cell lines tested after 24 h treatment by increasing concentrations of CHCl_3_ extract (Figure 4).

### 4.2. Human Studies

#### 4.2.1. Study Subjects

At the beginning of the study, 87 PC patients accepted to participate and regularly received green tea. After 6 months, 17 patients were excluded from the study (6 patients died during the study, 3 refused to continue the study after 3 months, and 8 did not drink tea or drank it only a few times during the entire period of the analysis). The cohort investigated consisted then in 70 PC patients with median age of 70.64 ± 6.5 years and median serum PSA level of 62.73 ± 33.97 ng/ml. The Gleason score at diagnosis was between 5 and 7 in 40% of cases and between 8 and 10 in 60% of cases. Eleven percent of cases underwent prostatectomy followed by hormone therapy, 11% underwent radiotherapy associated with hormone therapy, 49% were under hormone therapy, and 29% were under chemotherapy associated with hormone therapy. Controls were 85 age-matched healthy subjects which never or rarely drank green tea (median age 68.5 ± 6.56 years, *p* = 0.1169) and 35 age-matched healthy subjects who normally drank 1 to 3 cups of green tea per day for at least 1 year (median age 68.81 ± 6.42 years, *p* = 0.2537) (Table 3).

#### 4.2.2. Level of Lipid Peroxidation and Antioxidants Status

**Figure 5:**
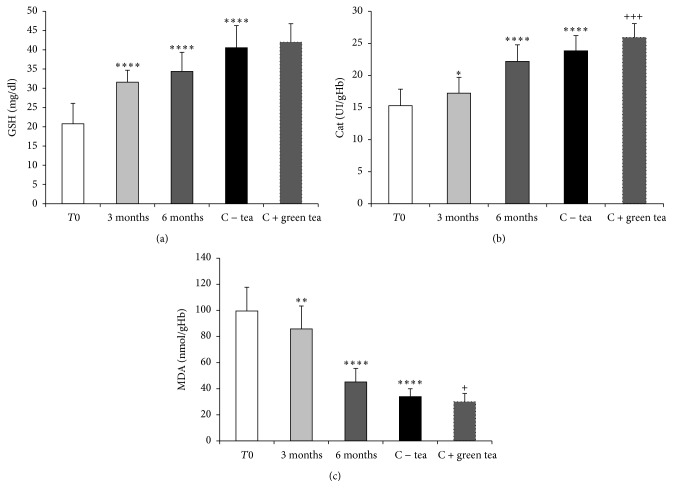
Effect of green tea consumption on lipid peroxidation and antioxidants status in erythrocytes from controls individuals and PC patients. (a) Effect of green tea consumption in erythrocytes GSH level in controls and PC patients. (b) Effect of green tea consumption in erythrocyte catalase activity in controls and PC patients. (c) Effect of green tea consumption in erythrocyte MDA level in controls and PC patients. *T*0: PC patients before green tea consumption; 3 months: PC patients after 3 months of green tea consumption; 6 months: PC patients after 6 months of green tea consumption; C − tea: control individuals who rarely or never drank tea; C + green tea: control individuals who usually drank 1 to 3 cups of green tea per day. Data are expressed as the means ± SD of three to four different experiments; ^*∗*^*p* < 0.05, ^*∗∗*^*p* < 0.01, and ^*∗∗∗∗*^*p* < 0.0001, compared to *T*0. ^+++^*p* < 0.005, ^+^*p* < 0.05, compared to C − tea.

To assess oxidative stress in erythrocytes from PC patients, lipid peroxidation and antioxidants were measured in erythrocytes before starting the green tea drinking protocol (*T*0). Significant alterations in reduced glutathione (GSH) level and in catalase activity were observed and a high level of MDA was found in PC patient's erythrocytes as compared to controls that never or rarely drank tea. GSH (mg/dl) was 20.79 ± 4.32 versus 40.51 ± 4.87 (*p* < 0.0001), CAT (UI/gHb) was 15.29 ± 1.75 versus 23.84 ± 2.03 (*p* < 0.0001), and MDA (nmol/gHb) was 99.52 ± 12.49 versus 33.83 ± 5.14 (*p* < 0.0001) (Figure 5). No significant difference was observed in GSH level between control individuals who regularly drank 1 to 3 cups of green tea per day and those that never or rarely drank tea (42.02 ± 4.23 mg/dl versus 40.51 ± 4.87 mg/dl), but catalase activity was higher (25.94 ± 1.81 UI/gHb versus 23.84 ± 2.03 UI/gHb, *p* < 0.005) and MDA level was lower (29.98 ± 4.73 nmol/gHb versus 33.83 ± 5.14 nmol/gHb, *p* < 0.05) in those individuals drinking green tea (Figure 5).

After three months of green tea consumption (2 to 3 cups per day), the levels of lipid peroxidation and antioxidants in erythrocytes of PC patients were evaluated again. Significant increases in GSH level (to 31.58 ± 2.57 mg/dl, *p* < 0.0001) and in catalase activity (to 17.23 ± 1.51 UI/gHb, *p* < 0.05) were observed and a significant decrease in MDA level was found (to 85.84 ± 12.05 nmol/gHb, *p* < 0.01) (Figure 5).

After another three months of green tea drinking, reduced GSH significantly increased to 34.36 ± 3.64 mg/dl, *p* < 0.0001 and catalase activity to 22.19 ± 1.78 UI/gHb, *p* < 0.0001. Furthermore, MDA level further decreased to 45.16 ± 7.45 nmol/gHb, *p* < 0.0001 (Figure 5).

## 5. Discussion

The excessive production of ROS, unbalance in antioxidant defense systems, or both combined may cause oxidative stress in different cell compartments, triggering cell signaling processes associated with initiation and development of many diseases including PC. Evidence suggests that PC progress is associated with increased oxidative stress [39]. Several possible mechanisms may contribute to increased ROS generation associated with PC development and progression including inflammation, diet, aging, NADPH oxidase, steroid hormones, and mitochondrial and nuclear DNA mutations [39]. A number of studies have investigated the effect of antioxidant supplements in preventing or reducing the risk of PC, although the results are still conflicting [40]. In the current study, we tested the capacity of green tea, described to have a general antioxidant property [10] to reduce oxidative stress in PC patients. A group of Algerian patients with PC were tested with a commercial Chinese green tea. For the safety of patients and to confirm the antioxidant/anti-proliferative activity of the Chinese green tea used, we first investigated its composition and its effects in vitro. The commercial green tea used was first extracted using different solvents (ChCl_3_, EtOAc, and *n*-BuOH), followed by evaluating phenolic and flavonoid content, as well as each extract antioxidant and antiproliferative activity. We confirmed here that the green tea used, namely, the extracts obtained, had significant antioxidant activity. Unlike the ChCl_3_ extract which contained very few amounts of phenols and flavonoids, the EtOAc and *n*-BuOH extracts significantly quenched the DPPH radical and inhibited lipid peroxidation. In addition, these two latter extracts showed a significant antiproliferative effect against the human PC-3 prostate cancer cell line. The simultaneous antioxidant and antiproliferative effects observed may be ascribed to their rich content of phenols and flavonoids [10, 41]. In fact, gallic acid [42, 43] and rutin [44] in both EtOAc and *n*-BuOH extracts and catechins [17, 45] in the EtOAc extract are present within significant amounts and are known for their antioxidant and antiproliferative activity. In fact, the literature demonstrates that often natural compounds can act as both antioxidants and anticancer agents, taking advantage of the particular redox environment and signaling pathways in cancer cells [46, 47]. Interestingly, all green tea extracts used did not show any cytotoxicity effect in the normal BJ fibroblasts, which was an indirect confirmation that the use of the green tea chosen was safe generally for patients.

Seventy PC patients and 120 age-matched healthy subjects participated in the current study. The evaluation of lipid peroxidation and the antioxidant markers in the subset of Algerian PC patient's erythrocytes at the beginning of the study showed the presence of increased oxidative stress, including increased MDA concentration and low levels of reduced glutathione (GSH) and catalase activity were measured when compared to the respective healthy controls. These results agree with many others studies [6, 8, 48] and confirmed that this oxidant-antioxidant imbalance may be one of the major factors responsible for PC development and progression in humans. A recent review with twenty three case-control studies focusing on the role of oxidative stress in PC patients also demonstrated increased oxidative stress profiles and impairment of antioxidant defense systems in PC patients, concluding that oxidative biomarkers MDA and 8OH-dg as well as antioxidant parameters SOD, CAT, GSH enzyme family, and vitamins C and E may be potentially predictive biomarkers of PC [49].

Some epidemiological studies found that green tea consumption decreases significantly the risk of PC in different populations. Consumption of 3 daily cups of green tea in southeast China and 5 or more cups in Japan significantly decreased the risk of PC in these two populations [14, 15]. However, these results are conflicting with other studies which did not show any association between green tea consumption and PC [48]. In the present study, we found that the consumption of 2 to 3 cups per day of green tea during 3 months can significantly increase the level of GSH and catalase activity and decreased the level of MDA in PC patient's erythrocytes. After 6 months of green tea consumption, results from PC patients became close to that of controls who never or rarely drank tea. Our data confirmed that the commercial green tea used, which showed a potent effect in our in vitro assays, decreased oxidative stress markers and improved the antioxidative status in erythrocytes from the PC population studied. Hypothesizing that erythrocytes may be an adequate proxy for the prostate tissue, it is likely that green tea antioxidant effects may prevent PC progression. These results are in line with the few studies performed so far in humans [22, 24]. The study of Bettuzzi et al. [22] was the first study showing that green tea catechins were safe and very effective for treating premalignant lesions before PC development. No significant change in PSA was observed but 33% of PC inhibition was observed in a group of volunteers with high grade prostate intraepithelial neoplasia (HGPIN) after 1 year of daily treatment with green tea catechins. The work by McLarty et al. supported a potential role of tea polyphenols in the treatment or prevention of PC. A significant decrease in serum level of prostate-specific antigen (PSA), hepatocyte growth factor (HGF), vascular endothelial growth factor (VEGF), insulin-like growth factor (IGF-1), and IGF binding protein-3 (IGFBP-3) was observed with no elevation of liver enzymes in men with PC after a short-term supplementation with daily doses of polyphenon E (a total of 1.3 g of tea polyphenols) [24]. Our findings offer complementary information about the efficiency of green tea for PC management in human through its ability to regulate oxidative stress observed in PC patients in our and in many other studies [6, 8, 48]. Improving the antioxidant status in PC patients may reduce exaggerated ROS production and consequently reduce PC progression. It was previously reported that ROS activate different signaling pathways including mitogen-activated protein kinase (MAPKs) and phosphoinositide-3-kinase (PI3K)/Akt [50]. These two signaling pathways were found to be overactivated in PC and are suggested to be involved in PC development and progression [51, 52]. The study of Kumar et al. indicated that ROS generation is directly proportional to aggressive phenotype of PC and that antioxidant therapy decreased Akt expression and modulated MAPKs activities, delaying the proliferation of PC cell lines [53].

In the present study, we also reenforced the positive effect of green tea consumption in oxidative stress in humans, by comparing lipid peroxidation and antioxidants in erythrocytes from controls that never or rarely drank tea to a group of healthy men that drink usually 1 to 3 cups of green tea per day for a long period of time. The results obtained showed that green tea consumption may also improve overall the antioxidative status in healthy men. This finding suggests that regular consumption of green tea may reduce the oxidative stress produced during life and theoretically decrease its negative effects.

These benefits of the green tea may be very interesting in the case of PC characterized by a long latency period. It is typically diagnosed in 50-year-old men or older [54], but in autopsy studies both PC and high grade intraepithelial neoplasia (HGPIN) are detected in the 3rd decade, showing a steady increase with age [55].

One limitation of the present study is that the effects of green tea in PC gland tissue were not directly measured. Whether the antioxidant/antiproliferative effects of green tea extracts observed in vitro also occur in the prostate tissue or whether the oxidative stress measured in erythrocytes is also observed in the prostate gland tissue from PC patients is not known. Still, several other studies used this same approach [56–58]. This was not possible due to ethical concerns and refusal of PC patients to suffer repeated biopsies. Further studies in PC patients are needed to determine the effect of the green tea consumption in the oxidative stress in PC gland.

## 6. Conclusion

The current study identified increased oxidative stress in erythrocytes from Algerian PC patients. More importantly, we demonstrated here a commercial green tea investigated rich in polyphenols and flavonoids and presenting potent antioxidant and anticancer activities in vitro significantly decreased oxidative stress markers in PC patients. Regular consumption of green tea for a long period may protect individuals from the negative consequences of oxidative stress produced during life.

## Figures and Tables

**Table 1:** The different phenols revealed in the green tea extracts and their levels (expressed as mg per kg of dry leaves of green tea).

Phenols	Phenolic content of green tea extracts (mg phenolic/kg plant)		
	CHCl_3_ extract	EtOAc extract	*n*-BuOH extract
Gallic acid	0.25	777.93	1344.96
Gentisic acid	0.15	ND	102.14
Catechins	0.04	174.95	ND
Chlorogenic acid	ND	2.37	21.18
4-Hydroxybenzoic acid	0.31	ND	1.8
Protocatechuic acid	0.03	2.49	3.51
Caffeic acid	0.04	7.43	5.66
Vanillic acid	5.00	46.31	20.04
4-Hydroxybenzaldehyde	0.3	0	ND
Rutin	0.11	22.2	646.29
*p*-Coumaric acid	0.70	15.00	4.18
Ellagic acid	0.51	0.85	1.35
Chicoric acid	ND	0.2	0.67
Ferulic acid	ND	ND	1.92
Hesperidin	ND	0.65	2.5
Apigenin 7-glucoside	ND	0	1.96
Rosmarinic acid	0.03	0.237	ND
Protocatechuic acid ethyl ester	ND	0.05	0
Salicylic acid	6.5	34.69	21.23
Resveratrol	ND	ND	0.08
Quercetin	0.71	ND	ND
Naringenin	0.08	0.35	ND
Kaempferol	ND	ND	0.43

ND: not detected.

**Table 2:** IC_50_ values of antioxidant activities of green tea extracts and vitamin C in DPPH and LPO assays, obtained from Figure 2 data. Values are mean ± SD (*n* = 3).

Extracts and standards	IC_50_ (*μ*g/ml)	
	DPPH	LPO
CHCl_3_	207.94 ± 3.12	—
EtOAc	2.98 ± 0.32	201.01 ± 2.55
*n*-BuOH	7.58 ± 0.74	302.18 ± 28.31
Vitamin C	5 ± 0.1	20 ± 1.06

**Table 3:** Patients and respective control data.

	Patients, *n* (%)	Controls-tea^1^, *n* (%)	Controls + green tea^2^, *n* (%)
*No. of subjects*	70	85	35
*Mean age ± SD*	70.64 ± 6.5	68.5 ± 6.56	68.81 ± 6.42
*p* value^a^		0.1169^b^	0.2537^c^
*Mean PSA ± SD (ng/ml)*	62.73 ± 33.97	1.43 ± 0.92	1.27 ± 0.97
*p* value^c^		<0.0001^b^	<0.0001^c^
*Gleason score*			
5–7	28 (40)	—	—
8–10	42 (60)	—	—
*Treatment*			
Prostatectomy + hormone therapy	8 (11)	—	—
Radiotherapy + hormone therapy	8 (11)	—	—
Hormone therapy	34 (49)	—	—
Chemotherapy + hormone therapy	20 (29)	—	—

^1^Control individuals who never or rarely drank tea; ^2^control individuals who usually drank 1 to 3 cups of green tea per day; ^a^based on unpaired *t* test; ^b^comparing PC patients to control individuals who never or rarely drank tea; ^c^comparing PC patients to controls who usually drank 1 to 3 cups of green tea per day; PSA = prostate-specific antigen.

## Additional Points

*Chemical Compounds Studied in This Article*. They are gallic acid** (**PubChem CID: 370); aluminum trichloride (PubChem CID: 24012); quercetin (PubChem CID: 5280343); 1,1-diphenyl-2-picrylhydrazyl (PubChem CID: 2735032); ascorbic acid (PubChem CID: 54670067); thiobarbituric acid (PubChem CID: 272368); penicillin (PubChem CID: 2349); streptomycin (PubChem CID: 19649); sulforhodamine B (PubChem CID: 9916275); 5,5′-dithiobis(2-nitrobenzoic acid) (PubChem CID: 6254).
